# Supplementary material for: Fuzzy ripple artifact in high resolution fMRI: identification, cause, and mitigation
Source: bioRxiv. 2024 Sep 9:2024.09.04.611294. Preprint. [Version 1] doi: 10.1101/2024.09.04.611294 (PMC11418939; doi:10.1101/2024.09.04.611294)
Supplement: Supplement 1 [file NIHPP2024.09.04.611294v1-supplement-1.pdf]

bioRxiv preprint doi: https://doi.org/10.1101/2024.09.04.611294; this version posted September 9, 2024. The copyright holder for this preprint (which was not certified by peer review) is the author/funder. This article is a US Government work. It is not subject to copyright under 17 USC 105 and is also made available for use under a CC0 license.

Supplementary information:

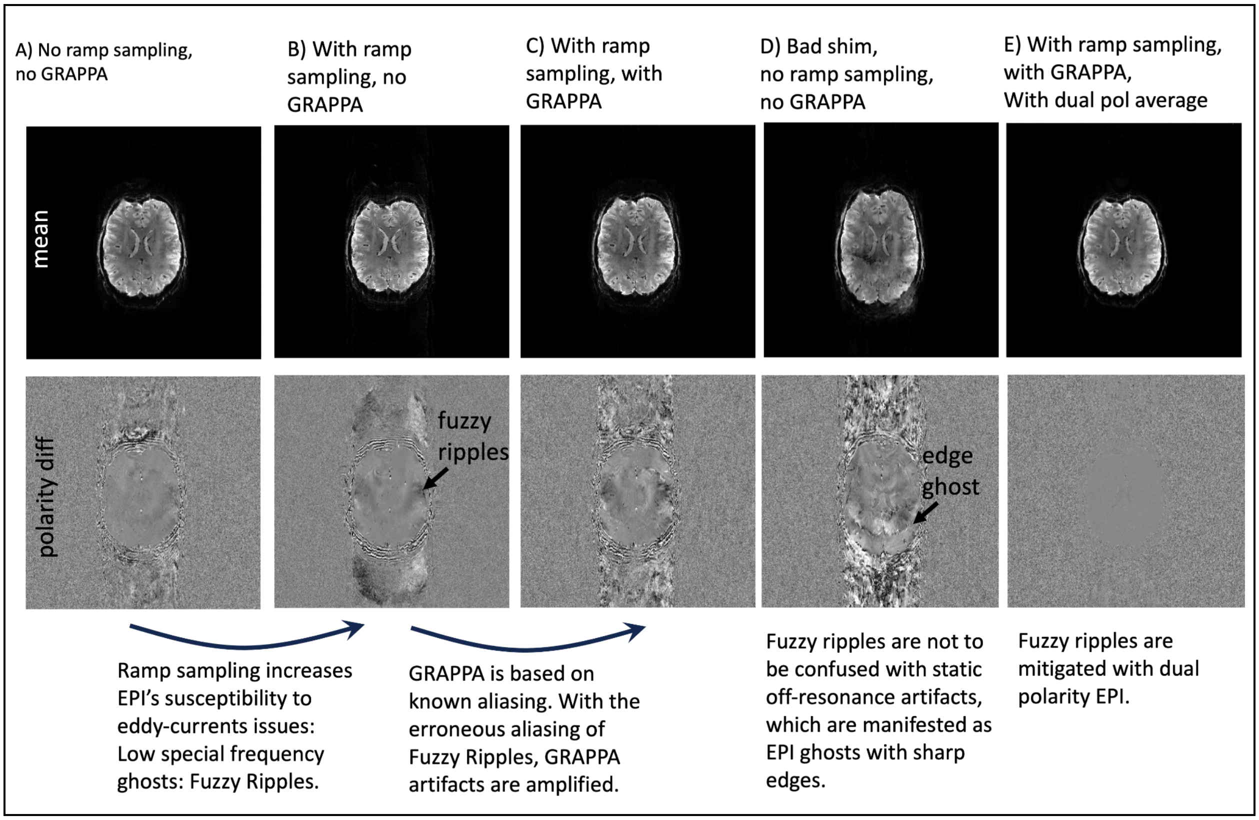

Figure S1: reproduced results of Fig. 4.: GRAPPA ghosts and static off-resonance ghosts.

A series of EPI acquisitions with various combinations of ramp sampling, bad B0 shim and GRAPPA are shown. The FOV purposefully chosen to be unconventionally large, to allow detections of the ghosts in the periphery. The signal difference between reverse EPI polarity images is shown to highlight the spatial ghost pattern that might be too weak to see with conventional image intensity windowing. The read direction in left-right, phase encoding direction is anterior-posterior.

A). *When EPI is done without ramp-sampling, imaging data are solely obtained during the flat top, which eliminates some parts of the largest gradient errors and, thus, the resulting EPI images only show relatively weak Fuzzy Ripples.*

B). *When ramp sampling is turned on, EPI becomes additionally sensitive to the largest peaks in gradient errors. Thus the Fuzzy Ripples become stronger. The Fuzzy Ripples manifest as aliasing of low spatial frequencies, as expected. Note that there are no sharp edges in the phase encoding direction.*

C). *Since, GRAPPA relies on a known aliasing pattern, which is contaminated with erroneous Fuzzy Ripples, it amplifies the effect.*

D). *This is different from static off-resonance effects. For example, in presence of suboptimal shimming (here purposefully altered), it does not amplify the low-spatial frequency fuzzy ripples. Instead, such settings add another source of artifact, namely the edge ghosts at high-spatial frequencies. These sharp borders look different from Fuzzy Ripples. Note that the edge is only sharp along the phase encoding direction. The Fuzzy Ripples that are also amplified with bad shim are smooth in the read directio,*

E). *The dual polarity approach can account for both of these sources of artifacts. The resulting images end up almost perfectly flat.*

Acquisition parameters of data presented here are mentioned in methods section 3.3.

bioRxiv preprint doi: https://doi.org/10.1101/2024.09.04.611294; this version posted September 9, 2024. The copyright holder for this preprint (which was not certified by peer review) is the author/funder. This article is a US Government work. It is not subject to copyright under 17 USC 105 and is also made available for use under a CC0 license.

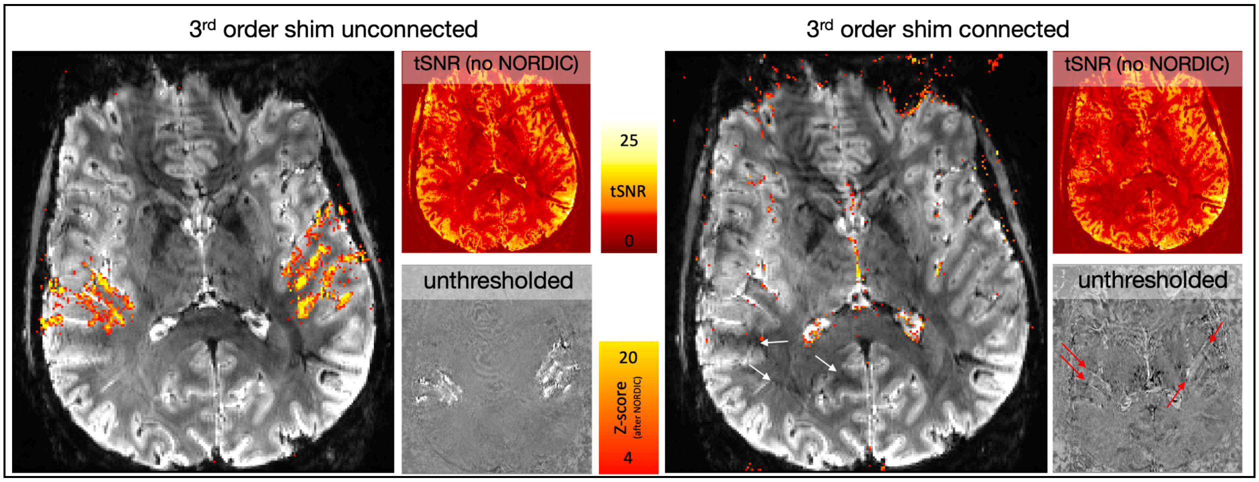

Figure S2: reproduced results of Fig. 5. How 3rd order shim induced Fuzzy Ripples affects fMRI activation detectability.

When the third order shim is connected, Fuzzy Ripples can be so strong that parts of auditory activation do not exceed the detection threshold.

White arrows point to Fuzzy Ripples that are stronger when scanning with the 3rd order shim connected. Fuzzy Ripples are still somewhat present without the 3rd order shim, they are weaker though.

Red arrows highlight activated brain areas. They are visible in unthresholded activation maps. However, they are below the detection threshold.

Acquisition parameters of data presented here are mentioned in methods section 3.4.

bioRxiv preprint doi: https://doi.org/10.1101/2024.09.04.611294; this version posted September 9, 2024. The copyright holder for this preprint (which was not certified by peer review) is the author/funder. This article is a US Government work. It is not subject to copyright under 17 USC 105 and is also made available for use under a CC0 license.

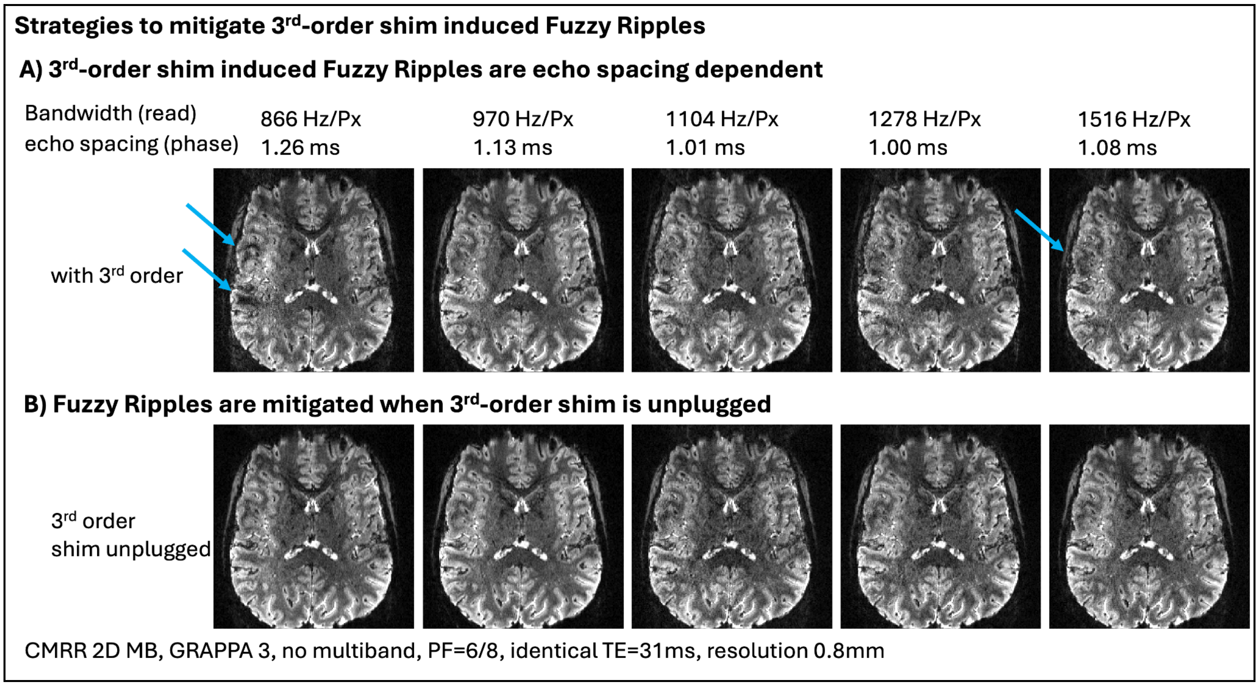

Fig. S3: reproduced results of Fig. 6 on a different scanner with a different participant using a different EPI sequence: 3rd-order shim induced Fuzzy Ripples as a function of echo spacing, cable connected, dual polarity averaging.

A.) The Fuzzy Ripple artifact is dependent on the echo spacing of the EPI readout. Thus, the artifact strength can be mitigated by protocol adjustments of the readout, which might come along with compromises of TE and readout efficiency.

B.) 3rd-order shim induces Fuzzy Ripples can be mitigated by means of unplugging its circuit. Breaking this circuit reduces the inductive coupling of the 3rd-order shim with the gradient.

Dual Polarity averaging results shown in Fig. 6C could not be reproduced with the CMRR sequence. Lacking access to the sequence source code, we could not make the required modifications to the sequence. For pilot tests of dual polarity averaging of this sequence in a phantom see (Huber 2023).

Acquisition parameters of data presented here are mentioned in methods section 3.5.

bioRxiv preprint doi: https://doi.org/10.1101/2024.09.04.611294; this version posted September 9, 2024. The copyright holder for this preprint (which was not certified by peer review) is the author/funder. This article is a US Government work. It is not subject to copyright under 17 USC 105 and is also made available for use under a CC0 license.

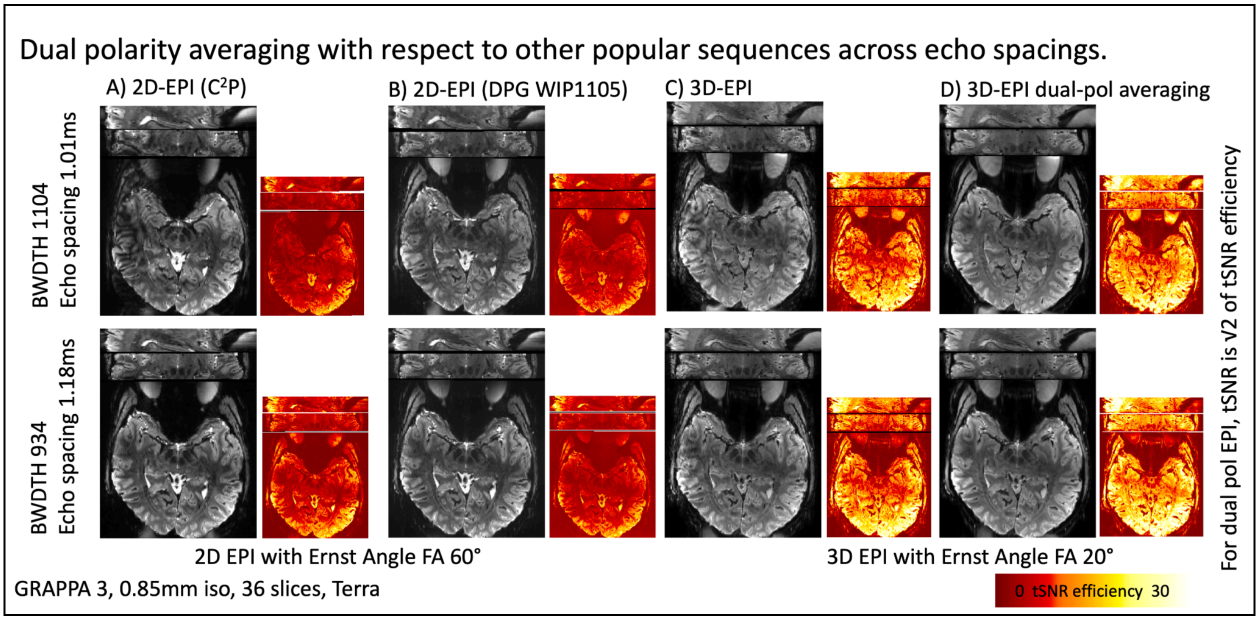

Fig. S4: reproduced results of Fig. 7 on a different scanner with a different participant with different echo spacings.

All sequences are used with the same resolution, and acceleration parameters. Echo spacing is constant for results in each row, respectively.

A) depicts the CMRR multiband sequence with these protocols. Fuzzy Ripple artifacts are visible with different strengths across echo spacings.

B) depicts the MGH simultaneous multi slice sequence of this protocol with its option of dual polarity GRAPPA. Fuzzy Ripple artifacts in the shorter echos spacing images are mitigated, but still visible.

C) depicts the same protocols with 3D-EPI. Due to its different M_z steady-state behavior, 3D-EPI has an inherently higher SNR. 3D-EPI suffers from Fuzzy Ripples, especially for shorter echo spacings.

D) depicts 3D-EPI with 3D-EPI with dual polarity averaging. It can be seen that Fuzzy Ripples are mitigated across echo spacings.

Acquisition parameters of data presented here are mentioned in methods section 3.6.

bioRxiv preprint doi: https://doi.org/10.1101/2024.09.04.611294; this version posted September 9, 2024. The copyright holder for this preprint (which was not certified by peer review) is the author/funder. This article is a US Government work. It is not subject to copyright under 17 USC 105 and is also made available for use under a CC0 license.

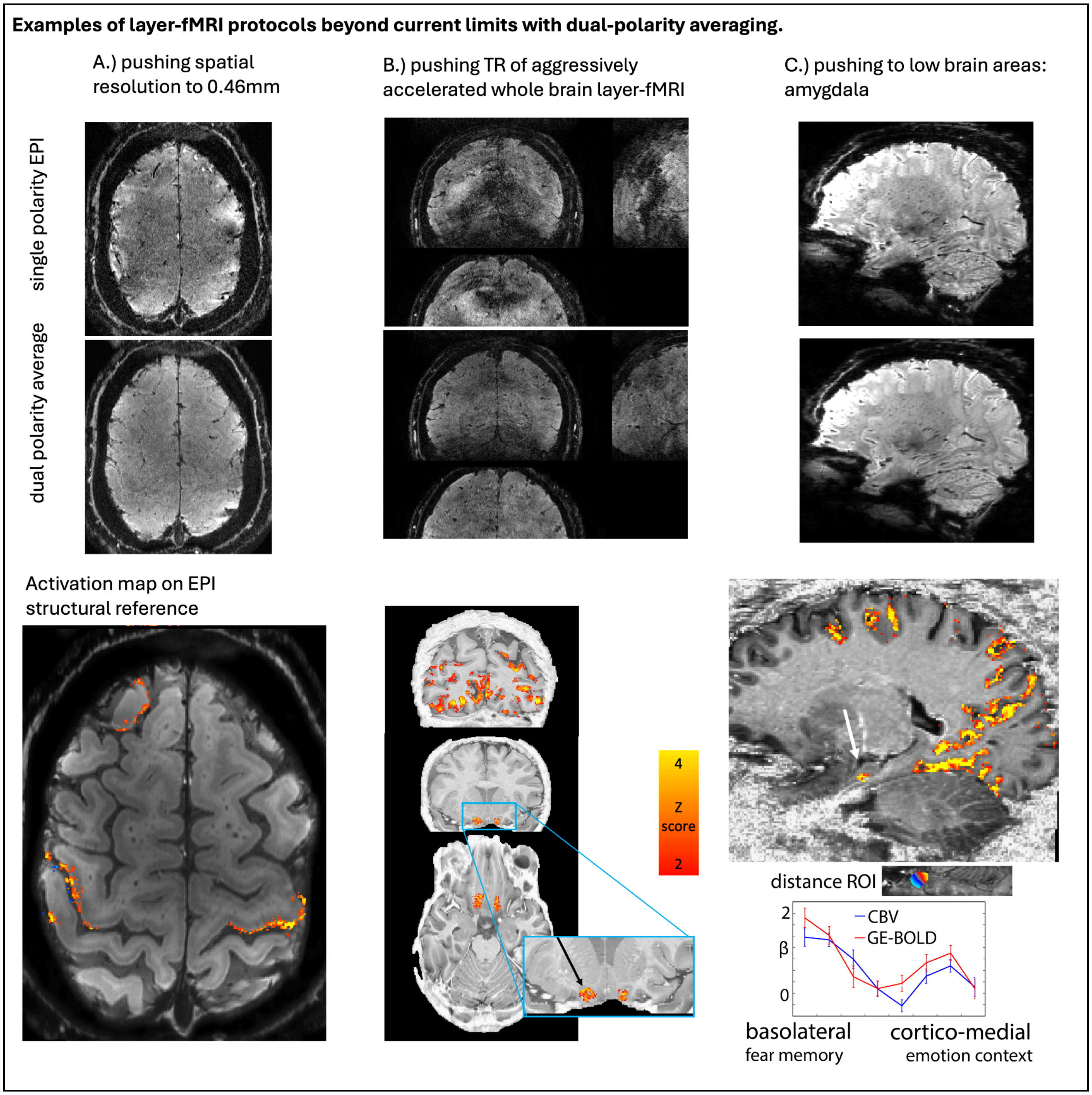

Fig. S5: reproduced results of Fig. 8: Examples of high resolution protocols pushing the limits of conventional protocols.

The individual panels show that pushing to high spatial resolution, to fast sampling (by means of acceleration), and to lower brain areas is challenged by Fuzzy Ripples (as shown in Fig. 1). Dual polarity averaging can mitigate these challenges, thus, allowing to overcome the current limits of conventional layer-fMRI protocols.

***A)** 7T Terra, 3D-EPI with GRAPPA 3, 6 fold segmentation, four runs of 12 min finger tapping, resolution of 0.46 mm.*

***B)** Feinbergatron 7T, 3D-EPI, GRAPPA 8, three 15-minute movie-watching sessions, resolution of 0.64 mm, TR=11.5 s for 314x314x180 voxels. Acquisition parameters of the data shown here are described in methods section 3.8.*

***C)** 7T Terra, 3D-EPI, GRAPPA 3, three times 15 min emotional faces vs. objects, resolution 0.82mm, Sagittal for deeper brain areas.*
